# Association of Thigh Muscle Strength with Texture Features Based on Proton Density Fat Fraction Maps Derived from Chemical Shift Encoding-Based Water–Fat MRI

**DOI:** 10.3390/diagnostics11020302

**Published:** 2021-02-13

**Authors:** Michael Dieckmeyer, Stephanie Inhuber, Sarah Schläger, Dominik Weidlich, Muthu R. K. Mookiah, Karupppasamy Subburaj, Egon Burian, Nico Sollmann, Jan S. Kirschke, Dimitrios C. Karampinos, Thomas Baum

**Affiliations:** 1 Department of Diagnostic and Interventional Neuroradiology, School of Medicine, Klinikum rechts der Isar, Technical University of Munich, Ismaninger Str. 22, 81675 Munich, Germany; sarah.schlaeger@tum.de (S.S.); egon.burian@tum.de (E.B.); nico.sollmann@tum.de (N.S.); jan.kirschke@tum.de (J.S.K.); thomas.baum@tum.de (T.B.); 2 Department of Sport and Health Sciences, Technical University of Munich, Georg-Brauchle-Ring 60, 80992 Munich, Germany; stephanie.inhuber@tum.de; 3 Department of Diagnostic and Interventional Radiology, School of Medicine, Klinikum rechts der Isar, Technical University of Munich, Ismaninger Str. 22, 81675 Munich, Germany; dominik.weidlich@tum.de (D.W.); dimitrios.karampinos@tum.de (D.C.K.); 4 VAMPIRE Project, Computing (SSEN), University of Dundee, Nethergate, Dundee DD1 4HN, UK; mrk2k2@gmail.com; 5 Pillar of Engineering Product Development, Singapore University of Technology and Design, 8 Somapah Road, Singapore 487372, Singapore; subburaj@sutd.edu.sg; 6 TUM-Neuroimaging Center, Klinikum rechts der Isar, Technical University of Munich, Ismaninger Str. 22, 81675 Munich, Germany; 7 Department of Diagnostic and Interventional Radiology, University Hospital Ulm, Albert-Einstein-Allee 23, 89081 Ulm, Germany

**Keywords:** magnetic resonance imaging, texture analysis, proton density fat fraction, thigh muscles, muscle strength

## Abstract

Purpose: Based on conventional and quantitative magnetic resonance imaging (MRI), texture analysis (TA) has shown encouraging results as a biomarker for tissue structure. Chemical shift encoding-based water–fat MRI (CSE-MRI)-derived proton density fat fraction (PDFF) of thigh muscles has been associated with musculoskeletal, metabolic, and neuromuscular disorders and was demonstrated to predict muscle strength. The purpose of this study was to investigate PDFF-based TA of thigh muscles as a predictor of thigh muscle strength in comparison to mean PDFF. Methods: 30 healthy subjects (age = 30 ± 6 years; 15 females) underwent CSE-MRI of the lumbar spine at 3T, using a six-echo 3D spoiled gradient echo sequence. Quadriceps (EXT) and ischiocrural (FLEX) muscles were segmented to extract mean PDFF and texture features. Muscle flexion and extension strength were measured with an isokinetic dynamometer. Results: Of the eleven extracted texture features, Variance(global) showed the highest significant correlation with extension strength (*p* < 0.001, R^2^_adj_ = 0.712), and Correlation showed the highest significant correlation with flexion strength (*p* = 0.016, R^2^_adj_ = 0.658). Multivariate linear regression models identified Variance(global) and sex, but not PDFF, as significant predictors of extension strength (R^2^_adj_ = 0.709; *p* < 0.001), while mean PDFF, sex, and BMI, but none of the texture features, were identified as significant predictors of flexion strength (R^2^_adj_ = 0.674; *p* < 0.001). Conclusions: Prediction of quadriceps muscle strength can be improved beyond mean PDFF by means of TA, indicating the capability to quantify muscular fat infiltration patterns.

## 1. Introduction

Thigh muscle composition and volume have been demonstrated to be affected by age, exercise, and a multitude of diseases, including musculoskeletal disorders, metabolic disorders, sarcopenia, and neuromuscular diseases [1,2,3,4,5,6,7,8,9,10,11,12,13]. In addition to tissue composition and volume, structure is hypothesized to play an important role for muscle quality and function, which has put it in the focus of interest as potential biomarker for the medical conditions mentioned above.

Magnetic resonance imaging (MRI), in particular quantitative MRI (qMRI), offers a non-invasive, radiation-free technique for the assessment of muscle tissue. It has been utilized for the evaluation of various properties, including cross-sectional area (CSA), fatty infiltration, inflammation, and structure. The proton density fat fraction (PDFF), measured by chemical shift encoding-based water–fat MRI (CSE-MRI) and validated by magnetic resonance spectroscopy (MRS) [14] and histology [15], has been established as a robust and reliable biomarker for muscle fat infiltration (MFI). In this context, mean PDFF values are commonly used.

Previous studies report on an inverse relationship between MFI and muscle function of the thigh [1,2,11,13,16,17,18]. Moreover, the relationship between mean PDFF and isometric strength of muscles has been investigated previously in the thigh and paraspinal region of healthy volunteers, demonstrating that mean PDFF better predicts muscle strength than CSA [18,19]. However, averaging PDFF values across a segmented muscle does not take into account the variation of muscle fat distribution within the region of interest (ROI), not fully capturing the available information on muscle structure and resulting muscle quality.

As a promising tool to reveal more quantitative information contained in medical imaging data, texture analysis (TA) has emerged in recent years [20,21]. Use cases include neurologic and oncologic imaging [22,23]. For example, TA based on multiple MRI sequences was shown to differentiate pathological and clinical subtypes of cervical carcinoma [24], and it improved the discrimination of normal and cancerous tissue of the prostate [25]. Regarding musculoskeletal imaging, TA has predominantly been performed on non-quantitative data, such as sonography [26], computed tomography [27], or conventional T2-weighted MRI sequences [28,29]. Furthermore, TA based on CSE-MRI-derived PDFF maps has been used in the quantitative analysis of vertebral bone marrow [30,31]. In this regard, PDFF-based TA can be considered superior in its ability to reveal information on muscular fat distribution and consequently differentiate muscles with different patterns of fatty infiltration.

Based on the hypothesis that muscle structure is a significant determinator of muscle function, the aim of our study was to investigate whether TA of thigh muscles improves the prediction of muscle strength beyond mean PDFF.

## 2. Materials and Methods

### 2.1. Subjects

30 healthy subjects (15 women, 15 men; age = 30.23 ± 5.97 years; body mass index (BMI) = 27.14 ± 2.60 kg/m^2^) were recruited for this study as outlined previously [32]. All subjects completed the International Physical Activity Questionnaire Short-Form (IPACQ-SF) ensuring a moderate level of physical activity and no history of high-performance sports [33,34]. Exclusion criteria were vertebral fractures, severe spinal anatomic abnormalities or pathologies (such as scoliosis and neuromuscular diseases), as well as general MRI contraindications. Informed written consent was obtained from all subjects for MRI examination and biometrical strength measurements. The study was approved by the local institutional committee for human research.

### 2.2. MR Imaging

All subjects underwent MRI on the same 3T system (Ingenia, Philips Healthcare, Best, Netherlands) using the built-in 12-channel posterior coil and a 16-channel anterior coil. Subjects were positioned head-first in a supine position. An axially prescribed six-echo 3D spoiled gradient echo sequence was used for chemical shift encoding-based water–fat separation covering the thigh region on both sides.

The sequence acquired the six echoes in a single TR using non-flyback (bipolar) read-out gradients with the following imaging parameters: TR/TEmin/ΔTE = 6.4/1.1/0.8 ms, field of view (FOV) = 220 mm × 401 mm × 252 mm, voxel size = 3.2 mm × 2.0 mm × 4.0 mm, frequency encoding direction = L/R, no SENSE, scan time = 1 min 25 s. A flip angle of 3° was used to minimize T_1_-bias effects [35].

The gradient echo imaging data was processed online using the vendor’s routines as described in the following: The multi-echo mDIXON algorithm performs a phase error correction followed by a complex-based water–fat decomposition using a precalibrated seven-peak fat spectrum and a single T_2_* to model the signal variation with echo time. The imaging-based PDFF maps were computed as the ratio of the fat signal over the sum of fat and water signals.

### 2.3. MR Image Segmentation

For each side, segmentation of the quadriceps (EXT) and ischiocrural (FLEX) muscles was performed by a radiologist by drawing ROIs on the PDFF maps, using the open-source software MITK (Medical Imaging Interaction Toolkit, German Cancer Research Center, Division of Medical and Biological Informatics, Heidelberg, Germany). The ROIs covered the ten most central slices of the thigh muscles and were generated semi-automatically by prescribing the ROI borders of slices 1, 4, 7, and 10 manually and generating the missing ROI slices by interpolation. Each generated ROI was visually checked and manually corrected if necessary, e.g., in case of apparent inclusion of subcutaneous adipose tissue or unintended exclusion of muscle tissue. Good reproducibility of this approach has been shown before [16].

**Figure 1:**
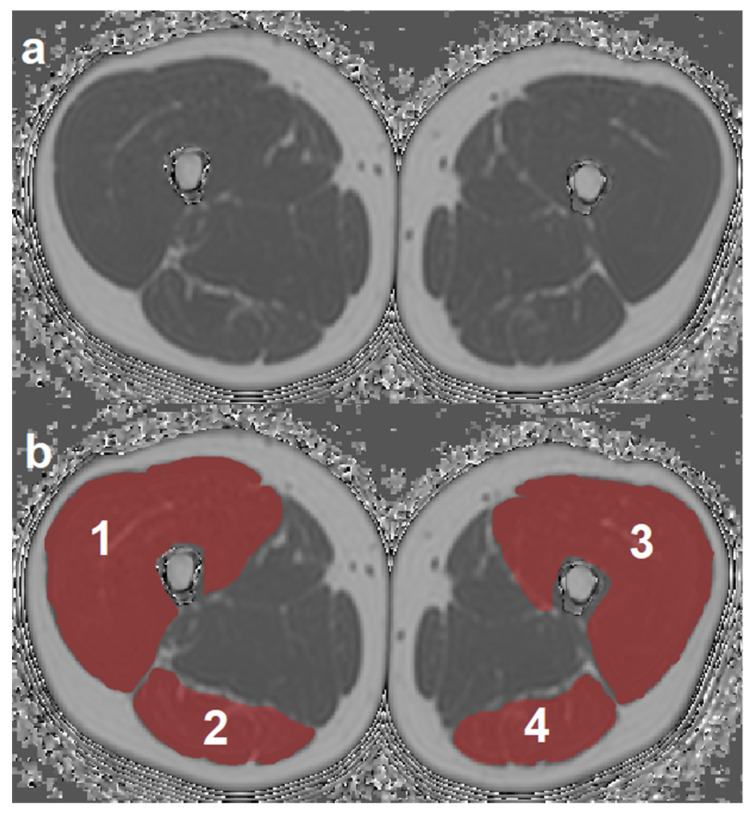
Representative axial PDFF map (**a**) with overlays of the four segmented muscle ROIs (**b**). **1**: right quadriceps muscle, **2**: right ischiocrural muscles, **3**: left quadriceps muscle, **4**: left ischiocrural muscles. (PDFF, proton density fat fraction; ROI, region of interest).

**Figure 2:**
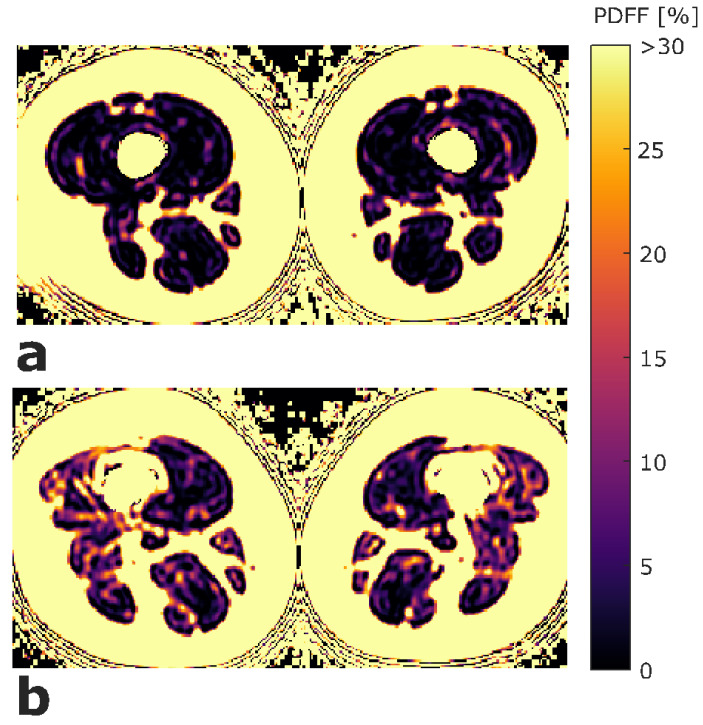
Exemplary color-coded axial PDFF maps of two study participants. (**a**) Participant with high strength (left side: MVIC_EXT_ = 281.7 Nm, MVIC_FLEX_ = 113.5 Nm; right side: MVIC_EXT_ = 298.7 Nm, MVIC_FLEX_ = 147.0 Nm) and low mean PDFF values (left side: PDFF_EXT_ = 1.07%, PDFF_FLEX_ = 1.46%; right side: PDFF_EXT_ = 0.31%, PDFF_FLEX_ = 0.98%). (**b**) participant with low strength (left side: MVIC_EXT_ = 126.7 Nm, MVIC_FLEX_ = 77.2 Nm; right side: MVIC_EXT_ = 149.1 Nm, MVIC_FLEX_ = 68.1 Nm) and high mean PDFF values (left side: PDFF_EXT_ = 5.31%, PDFF_FLEX_ = 6.94%; right side: PDFF_EXT_ = 4.71%, PDFF_FLEX_ = 8.59%). The upper limit of the color window was set to 30% to better depict the PDFF values within the thigh muscles. (PDFF_EXT/FLEX_, proton density fat fraction of quadriceps and ischiocrural muscles; MVIC_EXT/FLEX_, maximum voluntary isometric contraction of extension and flexion; Nm, newton meter).

ROIs were placed at the muscle contour to minimize the inclusion of subcutaneous fat or the muscle–fat interface. A representative PDFF map with corresponding segmentation masks of EXT and FLEX muscles is shown in Figure 1. Mean PDFF of each of the four muscles was extracted. Mean PDFF values were calculated of EXT and FLEX for the left and right side, respectively, to obtain values for both muscle groups on both sides (PDFF_EXT,left_, PDFF_EXT,right_, PDFF_FLEX,left_, and PDFF_FLEX,right_). Sample color-coded PDFF maps of two subjects, one with high strength and low mean PDFF, and one with low-strength and high-mean PDFF are shown in Figure 2.

### 2.4. Texture Analysis of PDFF Maps

Subsequent to segmentation, TA was performed on the PDFF maps of the segmented thigh muscles. Three global features (variance, skewness, kurtosis) and the following eight second-order features were extracted: energy, entropy, contrast, homogeneity, and correlation were calculated according to [36], variance and sum-average according to [37] and dissimilarity according to [38]. All texture features were calculated for each of the four muscles (EXT and FLEX for left and right side, respectively) to obtain texture feature values for both muscle groups on both sides (e.g., Variance (global)_EXT,left_, Variance_EXT,right_, Variance_FLEX,left_, and Variance_FLEX,right_).

Global features were extracted from intensity histograms. In histogram analysis, there is no universal method for choosing the ideal number and size of bins. The number of bins used in our analysis was calculated by taking the median of three different methods, known as Sturges’ method, Scott’s method, and the Freedman–Diaconis method since it yielded the most reasonable results compared to visual inspection of the histograms, and showed the best representation of the relevant data characteristics [39,40,41].

Second-order features were extracted using gray level co-occurrence matrix (GLCM) analysis [36]. As a preprocessing step, gray level quantization of the PDFF maps was performed to prevent sparseness by normalizing the image intensities using 200 equally sized bins and the minimum and maximum gray levels present, corresponding to values of 0% and 100%, respectively.

GLCM was obtained by computing the joint probability of two adjacent voxel intensities at a given offset *d* = (*dx*, *dy*, *dz*) and angular directions *θ* = (0°, 45°, 90°, and 135°). *dx*, *dy*, and *dz* denote the displacement along the *x*-, *y*-, and *z*-axis, respectively.

For 3D GLCM analysis, the co-occurrence probabilities of voxel intensities were computed from the 26 direct neighbors, aligned in 13 directions taking into account discretization length differences. The mean value of the features computed from the 13 directions ensures the rotation invariance. Image preprocessing, including isotropic resampling, gray level uniform quantization, and TA were performed using MATLAB 2018 (MathWorks Inc., Natick, MA, USA) and a radiomics toolbox (https://github.com/mvallieres/radiomics/ (accessed on 12 February 2021)) [42,43,44].

### 2.5. Isometric Muscle Strength Measurements

The maximum voluntary isometric contraction (MVIC) in single-joint knee extension and flexion was measured in Nm separately for each side using an isokinetic rotational dynamometer (IsoMed Back Module, D&R Ferstl GmbH, Hemau, Germany), as described previously [18,19].

### 2.6. Statistical Analysis

Statistical analyses were performed with SPSS 26.0 (SPSS Inc., Chicago, IL, USA) using a two-sided level of significance *α* = 0.05 for all statistical tests.

The Kolmogorov–Smirnov test indicated normally distributed data for age, BMI, PDFF, and isometric strength measurements, as well as for the majority of texture features. Mean and standard deviation (SD) of age, BMI, PDFF_EXT,left_, PDFF_EXT,right_, PDFF_FLEX,left_, PDFF_FLEX,right_, and texture features were calculated for males and females, and sex-dependent differences were compared using unpaired t-tests.

Multiple linear regression analyses were performed to determine significant correlations of the measured parameters and MVIC_EXT_. For each regression, independent variables were sex, side (left or right), age, BMI (all for adjustment), and one of the following twelve parameters: mean PDFF_EXT_ and texture features of the quadriceps muscles on both sides. Analogously, multiple linear regression analyses were performed to determine significant correlations of the measured parameters and MVIC_FLEX_. For each regression, independent variables were sex, side, age, BMI, and one of the following twelve parameters: mean PDFF_FLEX_ and texture features of the ischiocrural muscles on both sides. Adjusted R^2^ (R^2^_adj_) was calculated for each model.

Stepwise multivariate linear regression models were used to determine significant predictors of extension and flexion strength. Independent variables were sex, side, age, BMI, as well as PDFF_EXT_ and the eleven texture features for EXT (prediction of extension strength) or PDFF_FLEX_ and the eleven texture features for FLEX (prediction of extension strength). This resulted in 26 potential predicting variables, respectively. Inclusion (*p* < 0.05) and exclusion (*p* > 0.10) of independent variables in the linear regression models were based on the *p*-values of the F-test. Adjusted coefficient of determination (R^2^_adj_) was calculated for each model.

## 3. Results

Mean PDFF in the left and right quadriceps muscles was higher in males than females (left: 3.46% ± 1.51% vs. 3.15% 1.28%, right: 2.48% ± 1.68% vs. 2.31% ± 1.17%). Mean PDFF in the left and right ischiocrural muscles was lower in males than females (left: 3.44% ± 1.64% vs. 4.53% ± 1.71%, right: 3.16% ± 1.78% vs. 4.71% ± 2.43%). However, the differences were not significant (*p* > 0.05). There were no sex-dependent significant differences in age (males: 30.5 ± 4.9 years, females: 29.9 ± 7.0 years, *p* = 0.789) or BMI (males: 27.9 ± 3.1 kg/m^2^, females: 26.4 ± 1.8 kg/m^2^, *p* = 0.113).

Of the analyzed texture features, males showed significantly higher values than females for Variance(global) of the quadriceps muscles (left: 336.34 ± 45.26 vs. 284.61 ± 36.74, *p* = 0.002; right: 339.57 ± 44.02 vs. 289.64 ± 31.94, *p* = 0.001) as well as Variance(global) of the ischiocrural muscles (left: 151.20 ± 25.97 vs. 131.30 ± 23.86, *p* = 0.037; right: 152.61 ± 24.38 vs. 131.73 ± 23.91, *p* = 0.025). Males showed significantly lower values than females for Skewness(global) of the ischiocrural muscles (left: −0.73 ± 0.80 vs. −0.04 ± 0.72, *p* = 0.021; right: −0.54 ± 0.69 vs. 0.06 ± 0.89, *p* = 0.049).

On the left and right side, respectively, males showed significantly higher MVIC values than females for both extension (left: 236.92 ± 50.95 Nm vs. 146.57 ± 24.35 Nm, *p* < 0.001; right: 245.36 ± 36.75 Nm vs. 157.65 ± 27.73 Nm, *p* < 0.001) and flexion (left: 111.51 ± 19.15 Nm vs. 74.65 ± 12.28 Nm, *p* < 0.001; right: 113.71 ± 19.01 Nm vs. 69.54 ± 13.44 Nm, *p* < 0.001; Supplementary Table S1).

**Figure 3:**
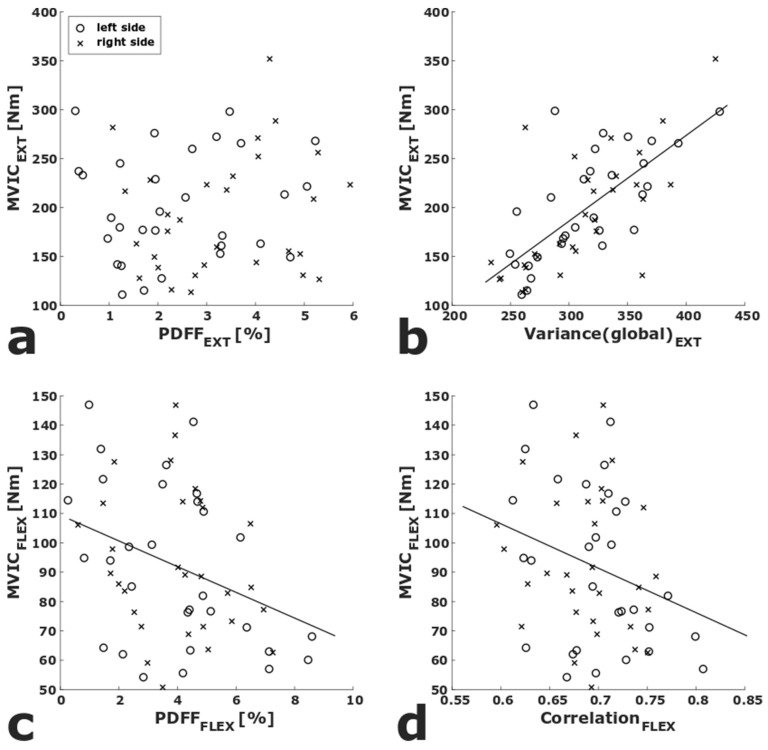
Scatter plots of PDFF_EXT_ vs. MVIC_EXT_ (**a**) and PDFF_FLEX_ vs. MVIC_FLEX_ (**c**). Scatter plots of the texture parameters with the highest R^2^_adj_ in the multiple adjusted linear regression analyses (Variance(global)_EXT_, Correlation_FLEX_) vs. MVIC_EXT_ (**b**) and MVIC_FLEX_ (**d**). Linear regression lines are displayed for significant correlations. (PDFF_EXT/FLEX_, proton density fat fraction of quadriceps/ischiocrural muscles; MVIC_EXT/FLEX_ maximum voluntary isometric contraction of quadriceps/ischiocrural muscles).

In the multiple linear regressions, adjusted for sex, side, age, and BMI, Variance(global)_EXT_ (*p* < 0.001, R^2^_adj_ = 0.712) and Variance_EXT_ (*p* = 0.038, R^2^_adj_ = 0.660) showed significant correlations with MVIC_EXT_. PDFF_FLEX_ (*p* = 0.009, R^2^_adj_ = 0.664), Skewness(global)_FLEX_ (*p* = 0.028, R^2^_adj_ = 0.652), and Correlation_FLEX_ (*p* = 0.016, R^2^_adj_ = 0.658) showed significant correlations with MVIC_FLEX_ (Table 1). To visualize the association of strength measurements with PDFF as well as the texture features with the highest R^2^_adj_, scatter plots of PDFF_EXT_ and Variance(global)_EXT_ vs. MVIC_EXT_, respectively, as well as PDFF_FLEX_ and Correlation_FLEX_ vs. MVIC_FLEX_ are shown in Figure 3.

In the multivariate linear regression analysis regarding extension strength (independent variables: sex, side, age, BMI, PDFF_EXT_, and the eleven texture features of EXT), sex (*p* < 0.001) and Variance(global)_EXT_ (*p* < 0.001) were identified as statistically significant predictors (R^2^_adj_ = 0.709; *p* < 0.001). In the multivariate linear regression analysis regarding flexion strength (independent variables: sex, side, age, BMI, PDFF_FLEX_, and the eleven texture features of FLEX), sex (*p* < 0.001), BMI (*p* = 0.001), and PDFF_FLEX_ (*p* = 0.008) were identified as statistically significant predictors (R^2^_adj_ = 0.674; *p* < 0.001). Age was not identified as a statistically significant confounder in any of the models.

## 4. Discussion

In our study, we showed that thigh muscle texture features, extracted from CSE-MRI-derived PDFF maps, significantly correlated with strength measurements of quadriceps and ischiocrural muscles. Compared to mean PDFF alone, texture features improved the prediction of muscle strength of the quadriceps but not of the ischiocrural muscles.

Several of the extracted texture features demonstrated significant sex-dependent differences: Variance(global) was higher in males than females in both quadriceps and ischiocrural muscles, and Skewness(global) was lower in males than females in the ischiocrural muscles. Inhuber et al. investigated sex-dependent differences of thigh muscle PDFF and CSA. In their study, females showed significantly higher mean PDFF of ischiocrural muscles on the right side, and males showed significantly higher CSA of quadriceps muscles on both sides and significantly higher CSA of ischiocrural muscles on the left side [18]. However, to date, no comparable studies have reported on the sex-dependence of MRI-based texture features of thigh muscles and future studies are needed to confirm our initial results in a broader age range.

In the last years, the application of texture features for advanced quantitative analysis of medical imaging has been growing, with an emphasis on oncology [45,46,47]. In a study with 41 female subjects, Burian et al. demonstrated that PDFF-based TA of vertebral bone marrow is feasible and can differentiate pre- and postmenopausal women [30]. To the best of our knowledge, CSE-MRI-based TA of thigh muscles has not been performed previously. Yet, the relationship between MRI-based measurements of muscle composition and isokinetic strength measurements has been investigated before. For thigh muscles, previous work shows that mean PDFF inversely correlates with isokinetic strength and improves the prediction of isokinetic strength beyond CSA [16,18]. This improved predictive power of mean PDFF was also demonstrated in paraspinal muscles [19]. The mentioned studies inspire the hypothesis that muscle composition is at least as important as pure muscle volume as a determinator of muscle function, and the two parameters CSA and mean PDFF may complement each other with respect to the prediction of muscle strength. Furthermore, in patients with neuromuscular disorders, an inverse relationship of thigh muscle PDFF and strength was shown [11,48]. The multivariate linear regression analyses performed in the present study revealed different results for the two analyzed thigh muscle groups. Sex and Variance(global)_EXT_, but not PDFF, were identified as significant predictors of extension strength, while sex, BMI, and PDFF_FLEX_, but none of the analyzed texture features, were identified as significant predictors of flexion strength. Hence, texture features improve the prediction of muscle strength beyond mean PDFF in the extensor compartment of the thigh. Based on the hypothesis that TA of muscle tissue derived from PDFF maps can quantify the distribution of muscle fat, this may be explained by a different pattern of MFI of the two muscular compartments, de facto meaning that TA can potentially differentiate quadriceps muscles with the same mean fat fraction but different pattern of fat infiltration, i.e., homogeneous vs. heterogeneous (e.g., in the form of fat streaks). However, we could not find a significant improvement of muscle strength prediction for the flexor muscles, which could have various explanations. In this context, differences between the two compartments regarding anatomy, such as number of involved joints (quadriceps: one joint vs. ischiocrural muscles: two joints), muscle volume and concomitant partial volume effects (quadriceps: high vs. ischiocrural: low volume; Figure 1), as well as physiology, such as neuromuscular activation (quadriceps: one peripheral nerve (N. femoralis) vs. ischiocrural: two peripheral nerves (N. tibialis, N. fibularis communis)) should be considered. The relatively small sample size of the study, potentially resulting in insufficient statistical power to demonstrate a significant effect for the flexor muscles, has to be acknowledged as well.

In the present study, TA of thigh muscles was performed based on quantitative PDFF maps. These maps were derived from CSE-MRI, which delivers sequences with fast acquisition and good reproducibility that can easily be added to routine imaging protocols of the thigh region [19]. Felisaz et al. applied TA in thigh musculature on non-quantitative T2-weighted spin-echo images for the purpose of machine-learning-aided prediction of water T2 and fat fraction [28]. However, TA has not been performed before on CSE-MRI-derived PDFF maps. For the analysis of tissue composition, CSE-MRI-derived PDFF maps have the advantage of higher inter- and intra-individual comparability as well as higher reliability, as compared to the analysis of non-quantitative MRI.

To the best of our knowledge, the present work is the first one applying TA based on qMRI of thigh muscles. We demonstrated an improved prediction of quadriceps muscle strength, as compared to mean PDFF. This is an encouraging finding, potentially implying that muscle quality, reflected by the pattern of muscular fat distribution, has a significant impact on extensor muscle function. Hence, the combination of TA and CSE-MRI, representing state-of-the-art qMRI acquisition and postprocessing methods, could help to discover novel insights into quality and function of thigh muscles.

There are limitations to the present study. First, the study cohort comprises only a rather small number of young and healthy subjects featuring a relatively low mean and narrow distribution of PDFF values. Regardless of the low variation in muscle fat content, we observed significant correlations of texture features and strength measurements in both thigh muscle compartments. This leaves room for the interpretation that even small changes in muscle composition are reflected by structural changes, which exert a relevant effect on the biomechanical function of the muscle. As a next step, future studies covering a broader age spectrum and relevant patient cohorts (e.g., neuromuscular, musculoskeletal, or metabolic disorders) are needed to (i) confirm our initial results, and (ii) further investigate the potential of CSE-MRI-based muscle TA in disease, thus deepening the knowledge of thigh muscle quality and (dys-)function. This could translate into improved detection of pathologic muscle alterations and MFI at an early stage of disease. Second, TA performed in the present study included only a limited number of texture features. Extending the TA through the inclusion of additional texture operators, such as rotationally invariant local binary patterns, could reveal even more information on muscle structure [49]. Third, the muscle ROIs were segmented as a whole, which means that both intra- and intermuscular adipose tissue (intraMAT, interMAT) contribute to the PDFF distribution and could consequentially affect the TA results. However, given the resolution and image quality of the acquired images (Figure 1), an accurate segmentation of the single components of the quadriceps and ischiocrural muscles and subsequent exclusion of intraMAT was not practical. As far as feasible with regard to signal-to-noise ratio and acquisition time, adjusting the MRI sequence parameters should be considered in future studies, to enable more detailed muscle segmentation excluding interMAT.

## 5. Conclusions

We demonstrated the feasibility of TA based on CSE-MRI-derived PDFF maps in thigh muscles. Our initial results show improved prediction of muscle strength beyond mean PDFF in the quadriceps muscle, possibly indicating an interaction between muscle function and fat distribution. Hence, PDFF-based TA may have the potential to distinguish quadriceps muscles based on the pattern of MFI and improve the detection and monitoring of muscular alterations.

## Figures and Tables

**Table 1:** Results of the linear correlation analyses (adjusted for sex, side, age and BMI) of proton density fat fraction (PDFF) as well as the analyzed texture features versus maximum voluntary isometric contraction (MVIC) in extension and flexion, respectively. Significant correlations are marked in bold and significant correlations after Bonferroni correction are marked with *. R^2^_adj_, adjusted coefficient of determination; *p*, *p*-value of the respective parameter based on the F-test.

Parameter	Extension		Flexion	
	R^2^_adj_	*p*	R^2^_adj_	*p*
**PDFF**	0.636	0.405	0.664	0.009
**Variance(global)**	0.712	<0.001 *	0.627	0.277
**Skewness(global)**	0.635	0.489	0.652	0.028
**Kurtosis(global)**	0.634	0.518	0.633	0.159
**Energy**	0.634	0.508	0.622	0.528
**Contrast**	0.649	0.103	0.623	0.432
**Entropy**	0.642	0.212	0.630	0.223
**Homogeneity**	0.640	0.264	0.630	0.214
**Correlation**	0.634	0.501	0.658	0.016
**SumAverage**	0.632	0.754	0.636	0.121
**Variance**	0.660	0.038	0.630	0.209
**Dissimilarity**	0.649	0.109	0.622	0.517

## Data Availability

The datasets generated during and/or analyzed during the current study are available from the corresponding author on reasonable request.

## Supplementary Materials

The following are available online at https://www.mdpi.com/2075-4418/11/2/302/s1, Table S1: Mean and standard deviation (SD) of proton density fat fraction (PDFF), analyzed texture features, and measured MVIC, separately for knee extensors (quadriceps) (EXT) and knee flexors (ischiocrural muscles) (FLEX) and grouped by sex (male, n = 15; female, n = 15). Significant differences (*p* < 0.05) between males and females are marked in bold.

## Abbreviations

BMI	body mass index
CSA	cross-sectional area
CSE-MRI	chemical shift encoding-based MRI
EXT	quadriceps muscles, extensors
FLEX	ischiocrural muscles, flexors
FOV	field of view
GLCM	gray level co-occurrence matrix
intraMAT	intramuscular adipose tissue
interMAT	intermuscular adipose tissue
IPACQ-SF	International Physical Activity Questionnaire Short-Form
L/R	left-right direction
MFI	muscle fat infiltration
MITK	Medical Imaging Interaction Toolkit
MRS	magnetic resonance spectroscopy
MVIC	maximum voluntary isometric contraction
MVIC_EXT_	MVIC of quadriceps muscle
MVIC_FLEX_	MVIC of ischiocrural muscles
Nm	newton meter
NMD	neuromuscular disease
PDFF	proton density fat fraction
PDFF_EXT,left_	PDFF of left quadriceps muscle
PDFF_EXT,right_	PDFF of right quadriceps muscle
PDFF_FLEX,left_	PDFF of left ischiocrural muscles
PDFF_FLEX,right_	PDFF of right ischiocrural muscles
qMRI	quantitative magnetic resonance imaging
ROI	region of interest
R^2^	coefficient of determination
R^2^_adj_	adjusted R^2^
SENSE	sensitivity encoding
T	Tesla
T1	longitudinal relaxation time
T2	transverse relaxation time
T2*	effective transverse relaxation time
TA	texture analysis
TR	time of repetition
TE	echo time
TE_min_	minimal echo time
ΔTE	echo time step
3D	three-dimensional

Texture features of the quadriceps muscle are abbreviated using the subscript _‘EXT’_, e.g., Correlation_EXT_. Texture features of the ischiocrural muscles are abbreviated using the subscript _‘FLEX’_, e.g., Correlation_FLEX_.
