# Supplementary material for: Association of Thigh Muscle Strength with Texture Features Based on Proton Density Fat Fraction Maps Derived from Chemical Shift Encoding-Based Water–Fat MRI
Source: Diagnostics (Basel). 2021 Feb 13;11(2):302. doi: 10.3390/diagnostics11020302 (PMC7918768; doi:10.3390/diagnostics11020302)
Supplement: Supplementary file 1 [file diagnostics-11-00302-s001.pdf]

diagnostics

Article

Association of Thigh Muscle Strength with Texture Features Based on Proton Density Fat Fraction Maps Derived from Chemical Shift Encoding-Based Water–Fat MRI

Michael Dieckmeyer [1,*], Stephanie Inhuber [2], Sarah Schläger [1], Dominik Weidlich [3], Muthu R. K. Mookiah [4], Karupppasamy Subburaj [5], Egon Burian [1,3], Nico Sollmann [1,6,7], Jan S. Kirschke [1,6], Dimitrios C. Karampinos [3] and Thomas Baum [1]

[1] Department of Diagnostic and Interventional Neuroradiology, School of Medicine, Klinikum rechts der Isar, Technical University of Munich, Ismaninger Str. 22, 81675 Munich, Germany; sarah.schlaeger@tum.de (S.S.); egon.burian@tum.de (E.B.); nico.sollmann@tum.de (N.S.); jan.kirschke@tum.de (J.S.K.); thomas.baum@tum.de (T.B.)
[2] Department of Sport and Health Sciences, Technical University of Munich, Georg-Brauchle-Ring 60, 80992 Munich, Germany; stephanie.inhuber@tum.de
[3] Department of Diagnostic and Interventional Radiology, School of Medicine, Klinikum rechts der Isar, Technical University of Munich, Ismaninger Str. 22, 81675 Munich, Germany; dominik.weidlich@tum.de (D.W.); dimitrios.karampinos@tum.de (D.C.K.)
[4] VAMPIRE Project, Computing (SSEN), University of Dundee, Nethergate, Dundee DD1 4HN, UK; mrk2k2@gmail.com
[5] Pillar of Engineering Product Development, Singapore University of Technology and Design, 8 Somapah Road, 487372 Singapore; subburaj@sutd.edu.sg
[6] TUM-Neuroimaging Center, Klinikum rechts der Isar, Technical University of Munich, Munich, Ismaninger Str. 22, 81675 Munich, Germany
[7] Department of Diagnostic and Interventional Radiology, University Hospital Ulm, Albert-Einstein-Allee 23, 89081 Ulm, Germany
* Correspondence: michael.dieckmeyer@tum.de; Tel.: +49-89-4140-4561; Fax: +49-89-4140-4563

Citation: Dieckmeyer, M.; Inhuber, S.; Schläeger, S.; Weidlich, D.; Mookiah, M.R.K.; Subburaj, K.; Burian, E.; Sollmann, N.; Kirschke, J.S.; Karampinos, D.C.; et al. Association of Thigh Muscle Strength with Texture Features Based on Proton Density Fat Fraction Maps Derived from Chemical Shift Encoding-Based Water–Fat MRI. *Diagnostics* **2021**, *11*, 302. https://doi.org/10.3390/diagnostics11020302

Academic Editor: Sven Nebelung

Received: 22 January 2021
Accepted: 11 February 2021
Published: 13 February 2021

Publisher's Note: MDPI stays neutral with regard to jurisdictional claims in published maps and institutional affiliations.

Copyright: © 2021 by the authors. Licensee MDPI, Basel, Switzerland. This article is an open access article distributed under the terms and conditions of the Creative Commons Attribution (CC BY) license (http://creativecommons.org/licenses/by/4.0/).

Table S1. Mean and standard deviation (SD) of proton density fat fraction (PDFF), analyzed texture features, and measured MVIC, separately for knee extensors (quadriceps) (EXT) and knee flexors (ischiocrural muscles) (FLEX) and grouped by sex (male, n = 15; female, n = 15). Significant differences ($p < 0.05$) between males and females are marked in bold.

Parameter	Male		Female		*p*
	Mean	SD	Mean	SD	
**Age [years]**	30.5	4.9	29.9	7.0	0.789
**BMI [kg/m^2]**	27.9	3.1	26.4	1.8	0.113
**PDFF**$_{EXT, left}$	3.46	1.51	3.15	1.28	0.084
**PDFF**$_{EXT, right}$	2.48	1.68	2.31	1.17	0.057
**PDFF**$_{FLEX, left}$	3.44	1.64	4.53	1.71	0.544
**PDFF**$_{FLEX, right}$	3.16	1.78	4.71	2.43	0.750
**Variance(global)** $_{EXT, left}$	336.34	45.26	284.61	36.74	0.002
**Variance(global)** $_{EXT, right}$	339.57	44.02	289.64	31.94	0.001
**Skewness(global)**$_{EXT, left}$	-0.77472	0.79763	-0.60771	0.74569	0.558
**Skewness(global)** $_{EXT, right}$	-0.77117	0.78302	-0.61101	0.73123	0.567
**Kurtosis(global)**$_{EXT, left}$	6.1126	1.6234	6.0131	1.2137	0.851
**Kurtosis(global)** $_{EXT, right}$	5.7449	1.5898	5.6851	1.0074	0.903
**Energy**$_{EXT, left}$	0.00233	0.00164	0.00216	0.00062	0.707
**Energy**$_{EXT, right}$	0.00180	0.00151	0.00152	0.00037	0.491
**Contrast**$_{EXT, left}$	139.39	43.17	132.65	25.07	0.605
**Contrast**$_{EXT, right}$	142.54	38.61	148.33	23.87	0.625
**Entropy**$_{EXT, left}$	10.197	0.530	10.185	0.337	0.941
**Entropy**$_{EXT, right}$	10.436	0.598	10.524	0.269	0.610
**Homogeneity**$_{EXT, left}$	0.32457	0.03967	0.31982	0.02384	0.694
**Homogeneity**$_{EXT, right}$	0.29668	0.04234	0.29194	0.01801	0.693
**Correlation**$_{EXT, left}$	0.66785	0.03279	0.66556	0.03663	0.858
**Correlation**$_{EXT, right}$	0.67012	0.03517	0.67093	0.03399	0.950
**SumAverage**$_{EXT, left}$	0.00237	0.00030	0.00223	0.00028	0.194
**SumAverage**$_{EXT, right}$	0.00237	0.00036	0.00227	0.00021	0.376
**Variance**$_{EXT, left}$	0.00520	0.00137	0.00498	0.00085	0.607
**Variance**$_{EXT, right}$	0.00536	0.00130	0.00565	0.00081	0.462
**Dissimilarity**$_{EXT, left}$	6.3276	0.9477	6.3293	0.6011	0.996
**Dissimilarity**$_{EXT, right}$	6.7383	1.0788	6.9069	0.5666	0.596
**Variance(global)**$_{FLEX, left}$	151.20	25.97	131.30	23.86	0.037
**Variance(global)**$_{FLEX, right}$	152.61	24.38	131.73	23.91	0.025
**Skewness(global)**$_{FLEX, left}$	-0.72612	0.79944	-0.04485	0.72210	0.021
**Skewness(global)**$_{FLEX, right}$	-0.53888	0.68579	0.05623	0.89315	0.049
**Kurtosis(global)**$_{FLEX, left}$	4.7946	2.3472	3.9210	1.2507	0.214
**Kurtosis(global)**$_{FLEX, right}$	4.3439	1.9087	3.9012	1.4120	0.476
**Energy**$_{FLEX, left}$	0.00111	0.00037	0.00109	0.00040	0.837
**Energy**$_{FLEX, right}$	0.00120	0.00046	0.00107	0.00021	0.340
**Contrast**$_{FLEX, left}$	213.62	42.24	194.74	30.04	0.169
**Contrast**$_{FLEX, right}$	201.45	29.13	184.90	26.03	0.112
**Entropy**$_{FLEX, left}$	11.000	0.384	11.073	0.351	0.591
**Entropy**$_{FLEX, right}$	10.963	0.458	11.055	0.264	0.505
**Homogeneity**$_{FLEX, left}$	0.26793	0.02528	0.26039	0.02216	0.393
**Homogeneity**$_{FLEX, right}$	0.27107	0.02961	0.26380	0.01326	0.393
**Correlation**$_{FLEX, left}$	0.67865	0.04887	0.69873	0.03854	0.222
**Correlation**$_{FLEX, right}$	0.68513	0.04441	0.71334	0.05416	0.130
**SumAverage**$_{FLEX, left}$	0.00252	0.00028	0.00219	0.00028	0.004

**SumAverage$_{FLEX, right}$**	0.00239	0.00023	0.00220	0.00027	0.049
**Variance$_{FLEX, left}$**	0.00839	0.00160	0.00817	0.00137	0.686
**Variance$_{FLEX, right}$**	0.00814	0.00164	0.00829	0.00163	0.810
**Dissimilarity$_{FLEX, left}$**	8.2982	0.8591	8.3651	0.7246	0.819
**Dissimilarity$_{FLEX, right}$**	8.1726	0.9328	8.1498	0.4629	0.933
**MVIC$_{EXT, left}$ [Nm]**	236.92	50.95	146.57	24.35	0.000
**MVIC$_{EXT, right}$ [Nm]**	245.36	36.75	157.65	27.73	0.000
**MVIC$_{FLEX, left}$ [Nm]**	111.51	19.15	74.65	12.28	0.000
**MVIC$_{FLEX, right}$ [Nm]**	113.71	19.01	69.54	13.44	0.000
